# One to one comparison of cell-free synthesized erythropoietin conjugates modified with linear polyglycerol and polyethylene glycol

**DOI:** 10.1038/s41598-023-33463-x

**Published:** 2023-04-19

**Authors:** Paria Pouyan, Anne Zemella, Jeffrey L. Schloßhauer, Ruben M. Walter, Rainer Haag, Stefan Kubick

**Affiliations:** 1 Institut for Chemistry and Biochemistry, Freie Universität Berlin, Takustr. 3, 14195 Berlin, Germany; 2 Fraunhofer Institute for Cell Therapy and Immunology (IZI), Branch Bioanalytics and Bioprocesses (IZI-BB), Am Mühlenberg 13, 14476 Potsdam, Germany; 3 Institute of Chemistry and Biochemistry-Biochemistry, Freie Universität Berlin, Takustr. 6, 14195 Berlin, Germany; 4 Institute of Biotechnology, Technische Universität Berlin, Gustav-Meyer-Allee 25, 13355 Berlin, Germany; 5 Faculty of Health Sciences, oint Faculty of the Brandenburg University of Technology Cottbus-Senftenberg, the Brandenburg Medical School Theodor Fontane and the University of Potsdam, Potsdam, Germany

**Keywords:** Biochemistry, Cell biology, Chemical biology, Molecular biology, Structural biology, Molecular medicine, Chemistry

## Abstract

With more than 20 Food and Drug Administration (FDA)-approved poly (ethylene glycol) (PEG) modified drugs on the market, PEG is the gold standard polymer in bioconjugation. The coupling improves stability, efficiency and can prolong blood circulation time of therapeutic proteins. Even though PEGylation is described as non-toxic and non-immunogenic, reports accumulate with data showing allergic reactions to PEG. Since PEG is not only applied in therapeutics, but can also be found in foods and cosmetics, anti-PEG-antibodies can occur even without a medical treatment. Hypersensitivity to PEG thereby can lead to a reduced drug efficiency, fast blood clearance and in rare cases anaphylactic reactions. Therefore, finding alternatives for PEG is crucial. In this study, we present linear polyglycerol (LPG) for bioconjugation as an alternative polymer to PEG. We report the conjugation of LPG and PEG by click-chemistry to the glycoprotein erythropoietin (EPO), synthesized in a eukaryotic cell-free protein synthesis system. Furthermore, the influence of the polymers on EPOs stability and activity on a growth hormone dependent cell-line was evaluated. The similar characteristics of both bioconjugates show that LPGylation can be a promising alternative to PEGylation.

## Introduction

EPO is known as the hormone that regulates the production of new red blood cells. Most of the recent publications deal with new findings about the mechanism of action of EPO on erythrocytosis^1^, ischemic stroke^2^, anemia^3^, hypoxia^4^, tumor angiogenesis^5^ and neurodegenerative diseases^6^. Genetically modified EPO variants in particular arise attention. Erythropoietin stimulating agents (ESA) were continuously improved starting with erythropoietin alpha and beta to variants with longer half-lives such as darbepoetin alfa^7^ and PEGylated EPO (PEG-EPO^8^). PEG-EPO is known to stimulate erythropoiesis more effectively at a prolonged dosing interval^9^. PEGylated EPO (Mircera®) got Food and Drug administration (FDA) approval in 2007 and is prescribed against anemia associated with kidney diseases^10,11^. Based on the success of PEGylated EPO and other biopharmaceutics, the worldwide market of PEGylated drugs is expected to reach 10.5 billion US dollars in 2024^12^. Nevertheless, an essential problem arises with the PEGylated biopharmaceutics: antibodies directed to PEG. PEG is seen as a non-toxic and non-immunogenic biocompatible polymer that is attached to more than 20 FDA-approved protein-based drugs^13^. In addition, non-protein therapeutics such as mRNA vaccines contain PEGylated nanoparticles^14^. The PEG-modified proteins, enzymes, peptides and nanoparticles are characterized by enhanced water solubility and proteolytic stability resulting in an extended half-life^15^. Although significant advancements has been made, PEGylated systems still have certain limitations that can restrict their widespread use. These limitations include the development of anti-PEG antibodies after repeated use of PEGylated substances, reducing their therapeutic effectiveness, and in specific cases, severe allergic reactions like anaphylaxis^16^. A study from Yang et al. identified anti-PEG-antibodies in 72% of contemporary samples by using a quantitative, competitive enzyme-linked immunosorbent assay^17^. Interestingly, they recognized that 50% of the serum samples from the 1970s to 1990s actually possessed anti-PEG-antibodies as well. Due to novel and more sensitive assays the detection of anti-PEG-antibodies and associated immunogenic reactions occur more frequently. In addition, the use of daily products such as cosmetics, soaps and medicines also containing PEG might also impact the presence of pre-existing anti-PEG-antibodies^18,19^. Therefore, a rethinking of handling PEGylated drugs is essential starting with a reliable detection of anti-PEG-antibodies and a precise adaption of therapies with PEGylated molecules. Moreover, many natural and synthetic polymers have been studied to replace PEG for half-life extension^20–23^. In this context linear polyglycerol (LPG) represents a promising candidate due to its similar structure and characteristics to PEG^24^. LPG and PEG both possess a polyether backbone, although, LPG carries a hydroxyl group on each repeating unit, which enables the introduction of immobilization, targeting, and labeling moieties^25,26^. LPG is highly biocompatible and esters of oligoglycerols with up to 10 repeating units have been approved by FDA as pharmaceutical and food additives and have been commercially available for a few decades^27,28^. LPG has recently been successfully conjugated to various model proteins via different conjugation chemistries, including random conjugation to bovine serum albumin^29^, site-specific conjugation via copper(I)-catalyzed azide-alkyne cycloaddition (CuAAC) to Exenatide, *N*-terminal conjugation to Interleukin^30^ and Anakinra^31^ and site-specific conjugation via strain-promoted azide-alkyne cycloaddition (SPAAC) to Interferon-α-2a^32,33^.

Although the coupling of PEGs with different molecular weights to EPO has been heavily evaluated in the last two decades, there are only a few reports demonstrating the use of alternative polymers. In addition, the peptide chain was often modified resulting in a deletion of glycosylation sites and the conjugation chemistry was limited to either *N*- or *C*-terminal^34^. For example, an erythropoietin mimetic peptide was coupled to a biodegradable hydroxyethyl starch, enhancing the biological effect^35^. Often these couplings are based on chemical processes with harsh conditions. In our study, we present for the first time a comparison of different polymers that were coupled by a biorthogonal click reaction under mild conditions to an EPO with all three *N*-glycosylation sites intact. In addition, using cell-free protein synthesis different EPO conjugates were synthesized within a very short time thereby enabling the analysis of various constructs in parallel. Therefore, we synthesized both site-specific PEGylated and LPGylated EPO and investigated their influence on EPO’s activity on the growth hormone dependent cell line TF-1. We have chosen the TF-1 cell line because of the presence of endogenous hEPO receptor. This cell line is known to proliferate in the presence of an active hematopoietic growth factor such as granulocyte–macrophage colony-stimulating factor (GM-CSF), different interleukins and EPO. We compared these LPG- and PEG-bioconjugates with marketed Mircera® and analyzed the individual EPO´s stability in human serum. For this purpose, EPO was synthesized in a cell-free system as previously reported^36^. The translationally active lysates contain endogenous ER-derived membrane vesicles, so called microsomes that enable a translocation into the lumen and subsequent posttranslational modification such as glycosylation and disulfide bridging^37^—both indispensable prerequisites for EPO’s activity. Since no *O*-glycosylation is possible in cell-free systems, this position was chosen for the coupling of the PEG- and LPG-polymers. Therefore, an amber stop codon was introduced into the gene sequence. Addition of an appropriate suppressor tRNA and an engineered aminoacyl-tRNA synthetase to the cell-free reaction led to the incorporation of an p-azido-l-phenylalanine (AzF) into the nascent polypeptide chain^36^. The azido group was further utilized to click cyclooctyne modified PEG and LPG via SPAAC, resulting in site-specifically modified EPO. Our study shows that LPGylated EPO conjugates show comparable activity to PEGylated counterparts in cell culture and were stable for more than 24 h.

## Material and methods

Anhydrous solvents (dimethylformamide and toluene), benzoylated cellulose dialysis tubes (2 kDa, 32 mm width), were purchased from Merck (Darmstadt, Germany). Tetra-n-octyl ammonium bromide 98% was purchased from ACROS Organics and used as received. All other chemicals were bought from Merck (Darmstadt, Germany) unless stated otherwise. 10 kDa α-methoxy-ω-amino-poly (ethylene glycol) (PEG-NH_2_) (Rapp POLYMERE, Tübingen, Germany) was used as received. ^1^H NMR spectra were recorded on a Bruker AMX 500 (Bruker Corporation) or JEOL ECP 500 (JEOL GmbH). Chemical shifts (δ) are reported in ppm via the deuterated solvent peak as the standard. IR measurements were done on a Nicolet AVATAR 320 FT_IR 5 SXC (Thermo Fisher Scientific) with a detector range of 4000–650/cm. Gel permeation chromatography (GPC) measurements in water were performed with an Agilent 1100 equipped with an automatic injector, isopump, and Agilent 1100 differential refractometer (Agilent Technologies, Santa Clara, CA, USA). The PSS Suprema (pre-column), 1 × with pore size of 30 Å, 2 × with pore size of 1000 Å (all of them with a particle size of 10 μm) column, was calibrated against Pullulan standards prior to measurements. The GPC measurements in tetrahydrofuran (THF) were done with an Agilent SECurity (1200 Series), equipped with an automatic injector, isopump, and UV and RI detector. The separation was done via a photoluminescence gel from Agilent (1 × pre-column, 3 × Mixed-C with a particle size of 5 μm), which was calibrated against polystyrene standards.

### Synthesis of cyclooctyne modified polyglycerol (LPG-BCN)

The acetal protection of glycidol, its polymerization, and chain-end modification was performed based on previously reported procedures from us^32^. In summary, in a flame dried Schlenck flask (Oct)_4_NBr (268 mg, 0.480 mmol, 0.008 eq), was dried under argon atmosphere. Thereafter, it was dissolved in 60 mL dry toluene at RT before addition of ethoxyethyl glycidyl ether (EEGE) (10 mL, 65.6 mmol, 1 eq). The mixture was cooled down in an ice bath and *i*-Bu_3_Al (2.1 mL, 2.4 mmol, 0.036 eq) was added fast under high-speed stirring. The reaction was let to run over night at RT and was quenched by addition of 1 mL ethanol. The crude product was purified by dialysis against acetone (MWCO: 2000 g/mol). The product was obtained as light-yellow oil (GPC in THF: Mn: 20687, Đ: 1.12). For the removal of acetal groups, the polymer was dissolved in ethanol (0.1 g/mL) and HCl (3% v/v ethanol) for at least two hours before dialysis in water (MWCO: 1000 g/mol). The product was obtained as a colorless oil (GPC water: Mn: 11885, Đ: 1.19). Thereafter, deprotected polymer (1 g, 0.08 mmol, 1 eq) was dissolved in 5 mL dry DMF and heated to 80 °C under reflux before addition of NaN_3_ (30 mg, 0.4 mmol, 5 eq) to the mixture. The reaction proceeded at this temperature for 3 days and purified via dialysis in water (MWCO: 1000 g/mol). The successful modification was observed by IR spectroscopy (2100/cm of azide peak). The azide group was reduced to amine by dissolving the LPG-N_3_ (1 eq) in water (0.05 g/mL). Afterwards, TCEP (1.5 eq to N_3_ groups) was added to the solution. The reaction was monitored by IR spectroscopy until the respective band of N_3_ disappeared. After that, the purification was done by dialysis in water (MWCO: 1000 g/mol). LPG-NH_2_ (1 eq) was then dissolved in dry DMF (32 mg/mL), then Et_3_N (3 eq) and BCN-NHS (1.5 eq) were added to the mixture. The reaction was stirred overnight and purified via dialysis against water (MWCO: 1000 g/mol). As LPG-modified BCN is prone to crosslinking in the dry state, the full conversion at this stage is assumed. Quality of LPG-BCN was monitored by ^1^HNMR spectrum (Supplementary Fig. 1). Based on GPC measurement in water, the LPG has a MW of 11.8 kDa.

### Synthesis of cyclooctyne modified polyethylene glycol (PEG-BCN)

The modification of commercial PEG-NH2 was done as the procedure described above for LPG-NH_2_. Quality of PEG-BCN was monitored by ^1^HNMR spectrum (Supplementary Fig. 2). Based on GPC measurement in water, the PEG has a MW of 10 kDa.

### Generation of template DNA

Gene sequence encoding the erythropoietin sequence (Uniprot P01588) was modified for cell-free protein synthesis and amber suppression. Therefore, the DNA template was changed as previously reported^38^. In addition, the native signal sequence was replaced by a melittin signal sequence (aaattcttagtcaacgttgcccttgtttttatggtcgtatacatttcttacatctatgcggac). A second construct was designed by exchanging the codon 153 (*O*-glycosylation site) to an amber stop codon. The sequences were manufactured by de novo synthesis (Biocat GmbH, Germany) and ligated into the pUC57-1.8 k vector backbone.

### Generation of orthogonal components

The engineered aminoacyl-tRNA synthetase (eAzFRS) and the suppressor tRNA were manufactured as described in detail in Zemella et al. 2019^39^. Briefly, the aminoacyl-tRNA synthetase was synthesized in the “RTS500 ProteoMaster *E. coli* HY Kit” (Biotechrabbit GmbH, Germany), purified via Strep-Tag, concentrated and stored at − 80 °C in a synthetase storage buffer (50 mM HEPES,10 mM KOAc, 1 mM MgCl2, 4 mM DTT, 0.02% NaN_3_, pH 7.6).

Template DNA for the suppressor tRNA was generated using a PCR reaction with a specific *O*-methyl primer pair, followed by run-off transcription. tRNA was purified by phenol–chloroform extraction using TRIzol-reagent (ThermoFisher Scientific), precipitated with isopropanol and resuspended in ultra-pure water. tRNA was folded in a PCR cycler (Biometra TRIO, Analytik Jena) and stored at − 80 °C.

### Cell-free synthesis of EPO

Cell-free protein synthesis was based on translationally active lysates derived from cultured *Spodoptera frugiperda* 21 (*Sf*21) cells. Lysate preparation was performed as described previously^40,41^. DNA template (60 ng/µL) was added to a reaction mixture composed of 20% (v/v) lysate, 100 µM amino acids, salts, energy components and PolyG (10 µM, biomers, Germany). A detailed protocol of the reaction is described in^38,42^. To monitor protein yield via scintillation counting and protein integrity via SDS-PAGE followed by autoradiography, uniformly radioactively labeled ^14^C-leucine (f.c. 30 µM, Perkin Elmer, Germany) was supplemented to the reaction. The ^14^C-leucine is statistically incorporated into the de novo synthesized protein. For the incorporation of the non-canonical amino acid 2 mM p-azido-l-phenylalanine, 3 µM eAzFRS and 5 µM suppressor tRNA were added. The successful integration was monitored by autoradiography. In detail, a coupled transcription-translation reaction using the plasmid containing an amber stop codon was performed in the presence and absence of orthogonal synthetase eAzFRS. The synthesis reaction in absence of eAzFRS results in a band pattern that correspondence to the EPO truncated at amino acid 153. This truncated EPO still possess all three *N*-glycosylations. The synthesis reaction in presence of eAzFRS results in a band pattern that correspondence to the full-length EPO with three *N*-glycosylations. A difference in band pattern—in particular a shift in the apparent molecular weight of EPO in presence of the synthetase verifies the incorporation of AzF. Additionally, the incorporation of AzF was previously shown by the specific coupling of an azide-reactive phosphine dye to EPO^36^.

The reactions were incubated for 3 h at 27 °C, 500 rpm, covered by an aluminum foil.

### Protein fractionation

Since EPO is translocated into the lumen of microsomal vesicles, the protein had to be released. Therefore, microsomes were centrifuged at 16,000 × g for 10 min at 4 °C, and the pellet was resuspended in PBS with the detergent *n*-Dodecyl-β-Maltoside (0.2% DDM, Sigma-Aldrich, St. Louis MO, USA), agitated for 45 min at 1000 rpm and centrifuged again. The supernatant was transferred into an empty Eppendorf tube and used for coupling of PEG- and LPG-polymers and for stability analysis as well as cell culture assays.

### Coupling of PEG- and LPG-polymers

Polymers were solved in water to a stock concentration of 5–10 mM. 10 ng cell-free produced EPO was incubated with 5 mM BCN-PEG and BCN-LPG in an Eppendorf tube over night at 4 °C. The coupling of the polymers was monitored by a shift of EPO in autoradiography.

### Deglycosylation assay

N-glycosylations of modified and unmodified EPO were cleaved by PNGase F (NEB, USA). The assay was performed according to the manufacturer´s protocol.

### Quantitative protein analysis

Protein yield of cell-free synthesized EPO was determined by hot trichloroacetic acid (TCA, Carl Roth GmbH, Germany) precipitation followed by liquid scintillation counting^43^.

### Qualitative protein analysis

EPO samples were precipitated in cold acetone, dried and resolubilized in LDS sample buffer (NuPAGE LDS sample buffer, Thermo Fisher Scientific). Samples were loaded onto precast SDS-PAGE gels (NuPAGE, 10% Bis–Tris, Thermo Fisher Scientific) and separated for 40 min at 180 V. Gels were washed with water, stained with simply blue safe stain (Thermo Fisher Scientific) and washed again. Afterwards gels were dried for 60 min at 70 °C (Unigeldryer 3545D, Germany). Gels were exposed to phosphor screens for 48 h. Bands were visualized by phosphor imaging (Amersham Typhoon RGB, GE Healthcare).

### EPO activity assay

TF-1 cells (Leibnitz-Institut DSMZ, Germany, DSMZ-No. ACC-334) expressing the hEPO receptor were cultivated in 85% RPMI-1640 (PAN Biotech), 13% FCS (Biochrom), 1% sodium pyruvate (Biowest), 1% penicillin–streptomycin (PAN Biotech) and 5 ng/mL GM-CSF (PeproTech, Germany). Cells were incubated at 37 °C and 5% CO_2_ in T25 and T75 flasks in a CO_2_ incubator (Binder, Germany). Cell density was kept between 2 and 7 × 10^5^ cells/mL. For the activity assay 2.0 × 10^5^ cells were transferred into fresh medium without GM-CSF. Each EPO variant (cell-free synthesized, recombinant EPO (Sigma-Aldrich, St. Louis MO, USA, #E5546-50 UG), Mircera® (Roche, Basel, Switzerland, 0.3 mL)) and GM-CSF were added with a final concentration of 10 ng/mL. An equal volume of a no template control (NTC) was added as negative control. Cell growth was monitored over one week by trypan blue staining in a Luna counting chamber (Logos biosystems). The assay was performed in triplicates and for each sample, two measurements were performed, resulting in six counted aliquots for each sample.

### EPO stability assay

EPO was synthesized as described above and coupled to PEG and LPG. Modified and non-modified EPO was incubated with human serum (SeraCon II, HiSS Diagnostics, Germany) for up to 24 h. After 0, 2, 4, 8 and 24 h an aliquot was taken and immediately frozen by liquid nitrogen. The aliquots were precipitated in acetone and loaded onto a SDS-PAGE. The integrity of the protein bands was analyzed by autoradiography.

## Results

### Cell-free synthesis of EPO and coupling of PEG-BCN and LPG-BCN

**Figure 1:**
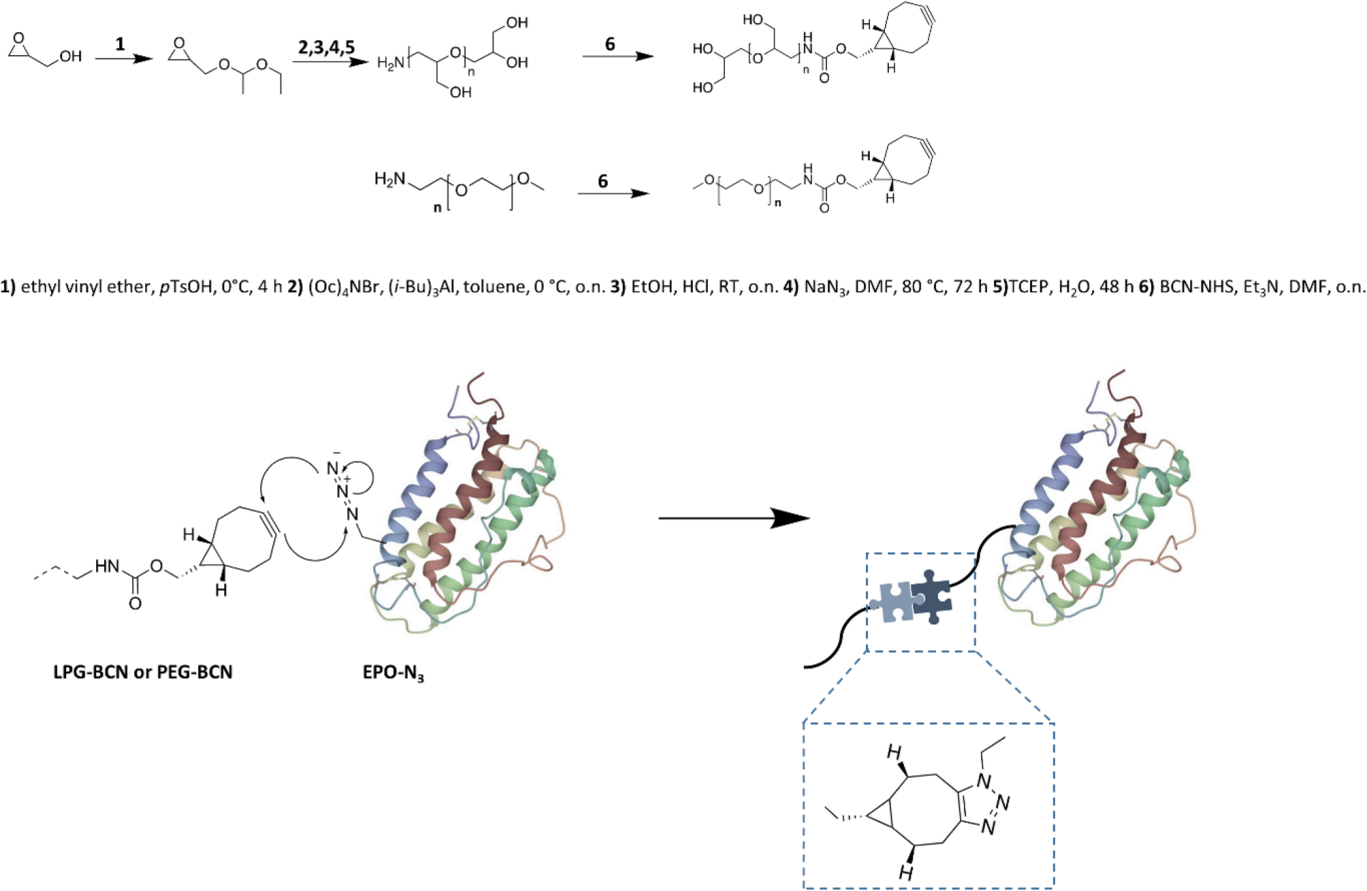
General scheme of the synthesis and chain-end modification of LPG and PEG with the following coupling to cell-free synthesized EPO. EPO structure obtained from PDB entry 1BUY^44^.

A general scheme of the synthesis and chain-end modification of LPG and PEG with the following coupling to cell-free synthesized EPO is seen in Fig. 1.

**Figure 2:**
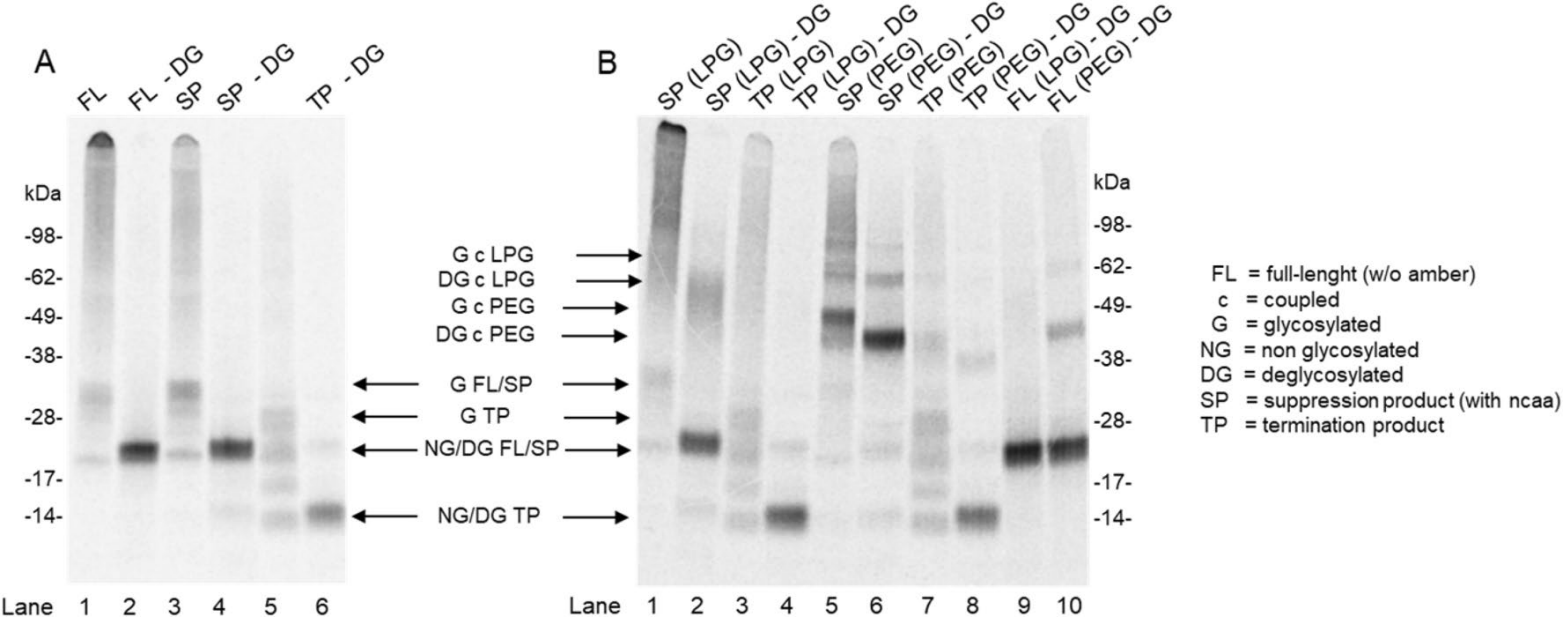
Chemoselective coupling of erythropoietin with LPG-BCN and PEG-BCN. (**A**) Autoradiography of different EPO variants: lane 1 and 2 full-length EPO without non-canonical amino acid, lane 3 and 4 suppression product with incorporated p-azido-L-phenylalanine, lane 5 and 6 termination product terminated at the amber stop codon. Each variant was analyzed in absence (lane 1, 3, 5) and presence (lane 2, 4, 6) of glycosidase PNGase F. Successful incorporation of AzF led to a comparable band pattern as seen for the full-length EPO without non-canonical amino acid. Synthesis of EPO (with amber stop codon) in absence of eAzFRS led to band pattern at a reduced molecular weight as expected for the truncated product. (**B**) Autoradiography after coupling of LPG-BCN and PEG-BCN to EPO. Lane 1–4 and lane 9 show the coupling of LPG-BCN to AzF containing EPO (lane 1–2), terminated EPO (lane 3–4) and full-length EPO (lane 9) in absence (lane 1 and 3) and presence (lane 2, 4, 9) of PNGase F. The successful coupling of LPG-BCN to AzF containing EPO is seen by a shift of EPO corresponding bands to a higher molecular weight (lane 1 and 2) at around 50–60 kDa. Lane 5–8 and lane 10 show the coupling of PEG-BCN to AzF containing EPO (lane 5–6), terminated EPO (lane 7–8) and full-length EPO (lane 10) in absence (lane 5 and 7) and presence (lane 6, 8, 10) of PNGase F. The successful coupling of PEG-BCN to AzF containing EPO is seen by a shift of EPO corresponding bands to a higher molecular weight (lane 5 and 6) at around 40–48 kDa. Uncropped autoradiography images are included in Supplementary information.

Cell-free protein synthesis of EPO resulted  in a distinct band pattern with four defined bands (Fig. 2A). These bands correspond to the three *N*-glycosylations of EPO (glycosylated at one, two or three sites) and the non-glycosylated form of EPO. The additional *O*-glycosylation of EPO is not addressed during cell-free protein synthesis. Therefore, the non-canonical amino acid was placed on the position of the *O*-glycosylation. The introduction of the non-canonical amino acid p-azido-l-phenylalanine (AzF) at the position of O-glycosylation did not alter the glycosylation pattern of EPO (azido-EPO, lane 3). The PNGase F glyco-digestion for both samples resulted in a lower band at the expected molecular weight of non-glycosylated EPO (lane 2 and 4). Without orthogonal synthetase applied during cell-free synthesis, no full-length EPO was visualized in the autoradiography (lane 5). Additionally, the glycosylated termination product was detected. After incubation with PNGase F, only the deglycosylated termination product was detected (lane 6).

Since the incorporation of AzF was verified in a first step, azido-EPO was incubated with BCN-modified PEG and LPG to obtain the site-specific bioconjugate (Fig. 2B). The incubation of azido-EPO with LPG-BCN resulted in a weaker band pattern at the expected molecular weight for uncoupled EPO. Additional, higher molecular smears and bands are visible, indicating a successful coupling of the polymer (Fig. 2B, lane 1). This assumption is verified by the incubation of the LPG-BCN with the truncated EPO resulting from termination at the amber stop codon. Here no additional bands were visible above the termination product for LPG-BCN (lane 3). Glyco-digestion of the LPG-EPO resulted in a shift of the smear to lower molecular weight, indicating that glycosylated EPO was coupled successfully to LPG-BCN (lane 2).

A similar result was obtained for the incubation of azido-EPO with PEG-BCN. Here defined bands at a higher molecular weight are visible, verifying the coupling of PEG-BCN to azido-EPO (lane 5). Again, after PNGase F-digestion, the prominent band shifted to a lower apparent molecular weight, indicating that glycosylated azido-EPO was coupled successfully to PEG-BCN (lane 6). In contrast to LPG-BCN, a slightly unspecific coupling to the termination product of PEG-BCN is detected by autoradiography (lane 7). This is even more visible by incubation of de-glycosylated EPO (without azido-group) with the polymers (lane 4 and 8). Here no coupling is expected since no AzF is present in the translated EPO. Therefore, a comparable band pattern to Fig. 1 lane 5 should be visible in the autoradiograph. Indeed, the same band pattern is visible in presence of LPG. In contrast, a slight additional band in presence of PEG is visible indication a slight unspecific binding of PEG to truncated EPO. A comparable unspecific coupling of PEG-BCN to full-length EPO without incorporated AzF was also detected (lane 10). No unspecific coupling of LPG-BCN to full-length EPO without incorporated AzF was observed (lane 9). Additional controls revealed the expected results (Supplementary Fig. 3) confirming the integration of AzF and the subsequent coupling of each polymer. Nevertheless, a mixture of LPG-coupled EPO and non-coupled EPO was obtained for cell culture analysis. Based on the autoradiography about 50–70% of the sample contains LPG-coupled EPO whereas the remaining 30–50% are composed of uncoupled EPO. Since the coupling of PEG to EPO resulted in a nearly 100% efficiency, we estimate that the percentage of PEG-coupled EPO for cell culture assays is around 90%. The remaining 10% are composed of uncoupled EPO and unspecific coupled PEG-EPO.

### Effects of PEG-EPO and LPG-EPO on cell culture

**Figure 3:**
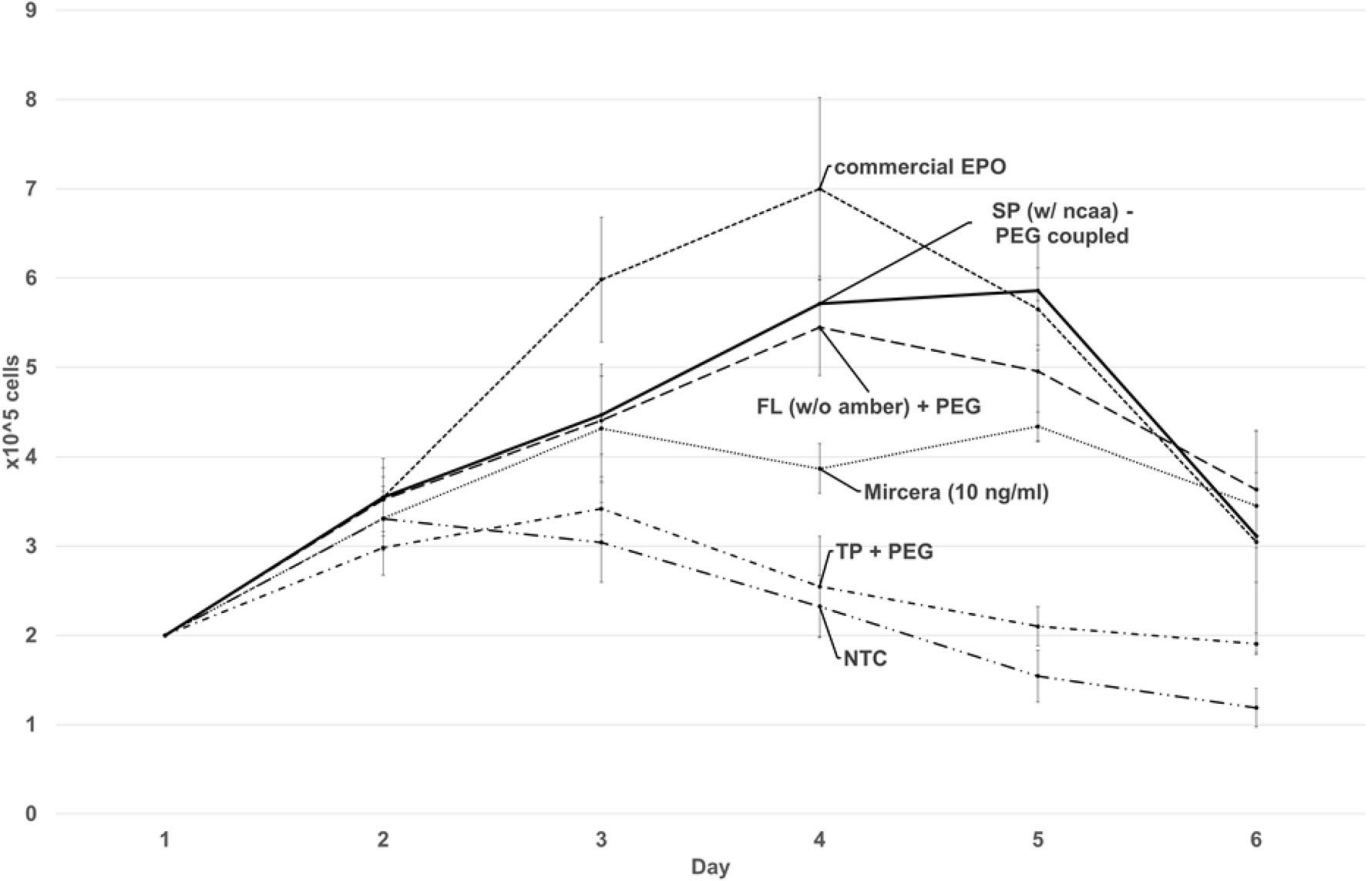
Cell-based activity assay of PEG-modified and non-modified cell-free synthesized EPO. Growth curves of TF-1 cell-line supplemented with PEG-modified EPO (continuous line), non-modified EPO (dashed line), termination product (dashed-single-dot line) cell-free no template control (NTC, dashed-double-dot line), commercial EPO (dashed line) and commercial Mircera® (grey dashed line). Concentrations of cell-free synthesized EPO variants were determined by TCA precipitation. 10 ng/mL of each sample was added to the TF-1 cells cultured in a 24-well-plate. Cells were counted for 6 days. Data are presented as the standard deviation of three independent experiments measured in duplicates (n = 3).

**Figure 4:**
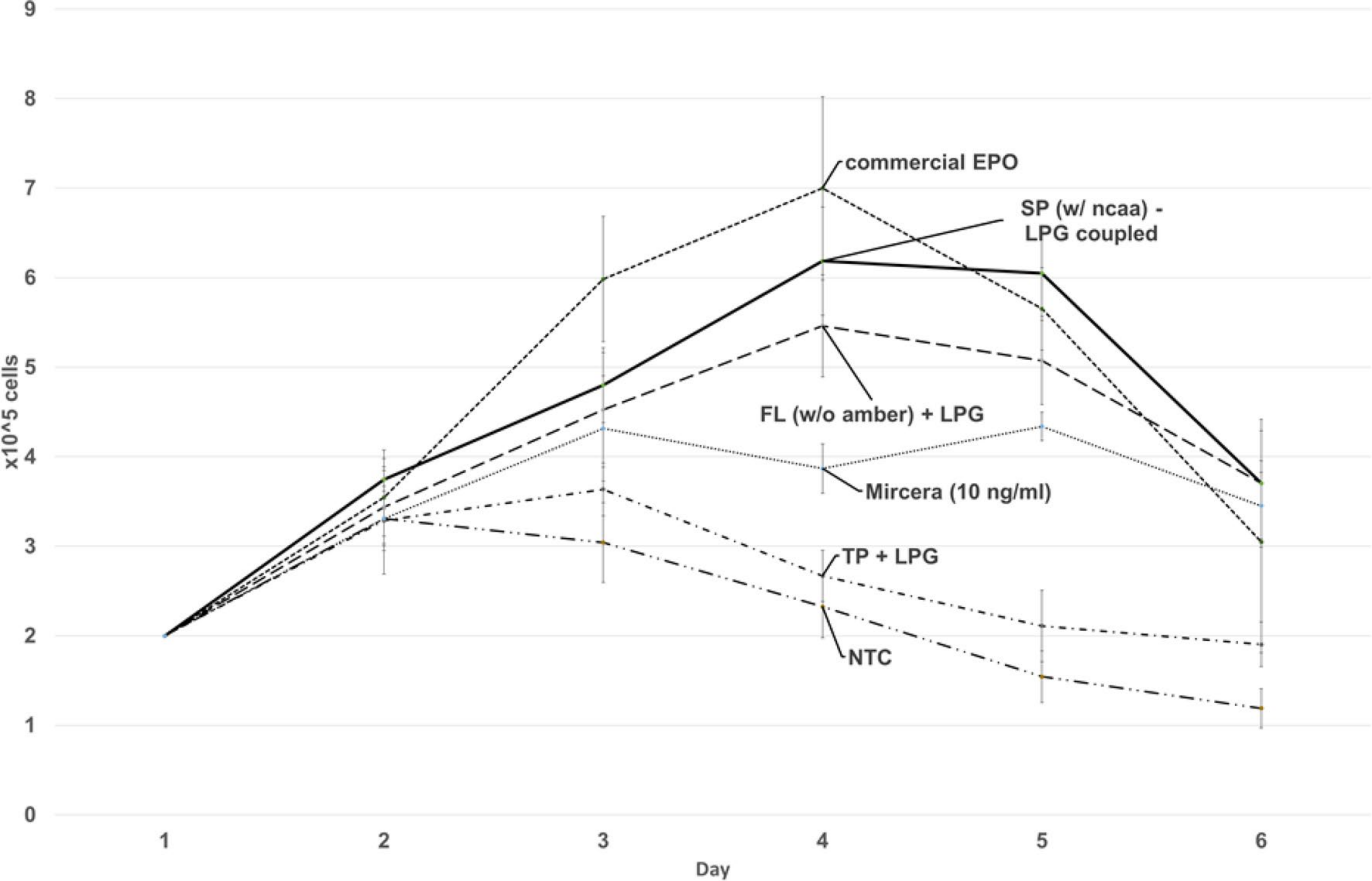
Cell-based activity assay of LPG-modified and non-modified cell-free synthesized EPO. Growth curves of TF-1 cell-line supplemented with LPG-modified EPO (continuous line), non-modified EPO (dashed line), termination product (dashed-single-dot line) cell-free no template control (NTC, dashed-double-dot line), commercial EPO (dashed line) and commercial Mircera® (grey dashed line). Concentrations of cell-free synthesized EPO variants were determined by TCA precipitation. 10 ng/mL of each sample was added to the TF-1 cells cultured in a 24-well-plate. Cells were counted for 6 days. Data are presented as the standard deviation of three independent experiments measured in duplicates (n = 3).

For the determination of activity of the individual EPO variants, a growth hormone dependent cell line (TF-1 cells) was cultivated in presence and absence of modified and non-modified EPO (Figs. 3 and 4). The activity assay showed that every sample containing EPO resulted in growth of cells comparable to commercially available EPO. The highest effect was seen after addition of PEG-EPO (Fig. 3). The effect was even higher compared to unmodified EPO and commercially available PEGylated EPO (Mircera®). As expected the controls (no template control and termination product) showed no or only limited activity (termination product). Interestingly the PEG-modified EPO and non-modified EPO showed nearly comparable growth curves until day 4. The growth curve of unmodified EPO reached its maximum after 4 days whereas the peak of PEG-modified EPO was calculated after 5 days. Moreover, cells incubated with PEG-modified EPO showed a prolonged growth curve.

A comparable effect was seen after the addition of LPG-EPO and the corresponding controls (Fig. 4). The highest growth rate was determined after addition of commercial EPO resulting in a maximum cell density of 7 × 10^5^ cells/mL. Again, the effect of LPG-EPO with a maximum cell density of 6.2 × 10^5^ cells/mL was comparable to commercial EPO. Strikingly, the maximum of counted cells after addition of LPG-EPO was in agreement with PEG-EPO at day 4 and 5. The effect of LPG-EPO was even higher in comparison to the addition of PEG-EPO. Again, addition of cell-free synthesized non-modified EPO resulted in a maximum cell density at day 4 with 5.5 × 10^5^ cells/mL.

The expected results were detected for the controls: Cells die quickly in presence of termination product and NTC. Additional controls did not revealed any effect of the polymers on cell culture (Supplementary Fig. 4).

### Stability analysis of PEG- and LPG-EPO

**Figure 5:**
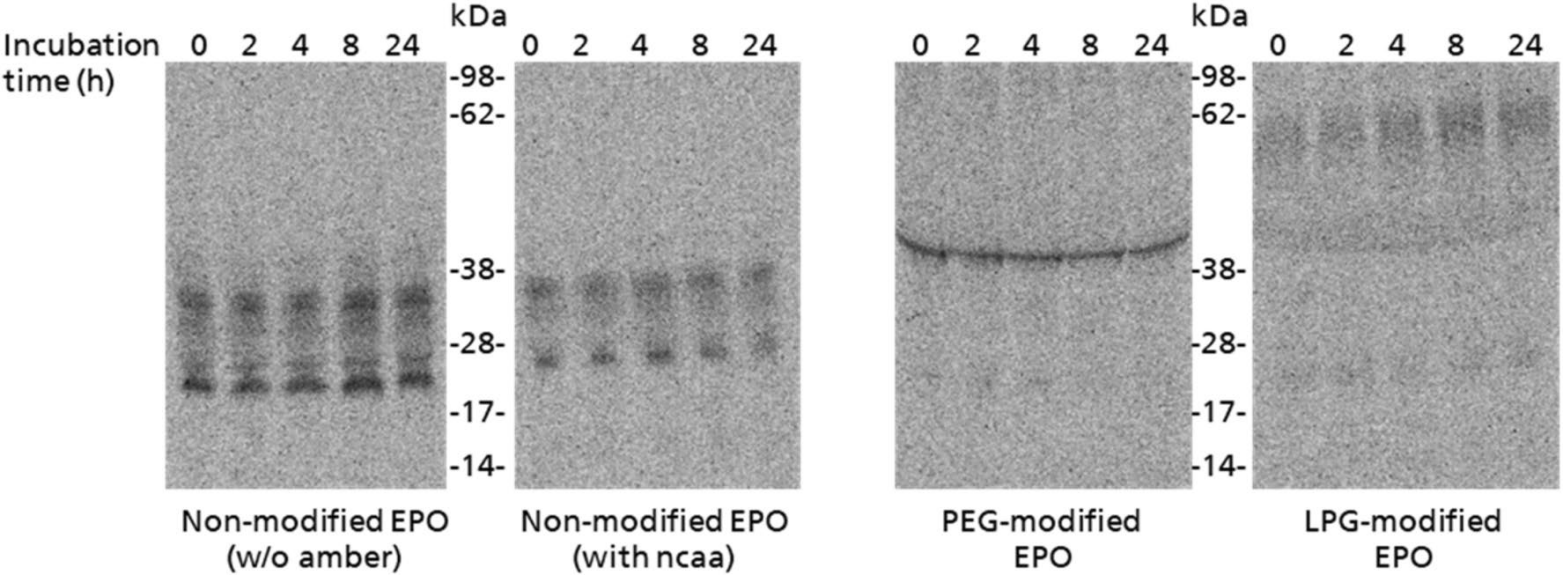
Stability analysis of modified and non-modified EPO. Unmodified full-length EPO, unmodified EPO harboring a non-canonical amino acid (ncaa), PEG-modified and LPG-modified EPO were incubated up to 24 h in human serum. Afterwards samples were acetone precipitated and analyzed via SDS-PAGE with following autoradiography. No change in band pattern was seen after 24 h indicating stable EPO samples. Uncropped autoradiography images are included in Supplementary information.

The effect of conjugated LPG and PEG on EPO’s stability was further analyzed by a serum stability test (Fig. 5). Modified and non-modified EPO variants were incubated for 24 h in human serum. Samples were collected at predetermined time points. The protein integrity was analyzed by autoradiography. Non-modified EPO (full-length and with non-canonical amino acid) showed the characteristic band pattern consisting of non-glycosylated EPO and additional, EPO that is glycosylated at up to three sites. PEG-EPO showed only one band at a higher molecular weight than EPO, corresponding to the polymer coupled protein. No uncoupled protein was detected. LPG-EPO showed again a smear above the band of PEG-EPO. Slight bands for uncoupled EPO are visible. Conjugation of PEG and LPG showed no significant influence on EPOs serum stability, as all bioconjugates showed a comparable band pattern even after 24 h of incubation in human serum.

## Discussion

Poly (ethylene glycol) (PEG) has been the “gold standard” to improve protein characteristics such as stability, solubility and immunogenicity. A wide range of different PEG structures were manufactured during the past 30 years ranging from small molecular weights (550 Da) to large linear and branched polymers (> 8,000,000 Da)^45^. Whereas in 1992 only seven different PEG structures in cosmetic products were detected^46^, this number increased to over 340 different PEG structures in 2015^47^. The frequent usage of PEG is also related to the inert and non-immunogenic behavior of PEG. Nevertheless, recent reports show that anti-PEG antibodies can occur after injection of PEGylated liposomes, proteins^48,49^ and after contact to cosmetic products containing PEG. In particular, multiple short PEG chains (< 10 kDa) attached to proteins have a higher probability to induce anti-PEG antibodies^50,51^. A recent study has analyzed the impact of pre-existing anti-PEG IgM and IgG antibodies on the therapeutic efficacy of PEGylated EPO^52^. For this purpose, a mouse model with pre-injected anti-PEG monoclonal antibodies was established before PEG-EPO administration. As a result, the ability of PEG-EPO to induce production of new red blood cells was blocked by anti-PEG antibodies due to accumulation of PEG-EPO in liver and spleen. The biological activity of PEG-EPO was recovered by increasing the initial concentration. Therefore, the appropriate dose of PEG-EPO should be evaluated dependent on a measurement of pre-existing anti-PEG antibodies in individual patients. Alternatively novel biomolecules that have a similar function as PEG might circumvent current problems with anti-PEG antibodies.

Meanwhile alternative synthetic polymers such as poly(glycerols), poly(oxazolines), poly(hydroxypropyl methacrylate), poly(2-hydroxyethyl methacrylate), poly(*N*-(2-hydroxypropyl) methacrylamide), poly(vinylpyrrolidone), poly(*N*,*N*-dimethyl acrylamide), and poly(*N*-acryloylmorpholine) have been studied to replace PEG^53,54^. Each of the polymers displays unique advantages such as non-immunogenicity, high hydrophilicity, good biocompatibility but also disadvantages such as accumulation, non-biodegradability and partly high synthesis costs^55^. Therefore, polymers have to be evaluated in detail to gain information about the individual protein´s characteristics and its possible impact on the human body.

Due to the structural characteristics of linear polyglycerol (LPG), it proposes a promising candidate for conjugation to proteins. In a study comparing the PEG- and LPGylated liposomes, Abu Lila et al. observed that in contrast to PEGylated liposomes, modification with LPG enhances the in vivo performance of the system. LPGylated liposomes did not induce accelerated blood clearance (ABC), a limitation of PEGylated liposomes upon repeated administration, which can negatively influence the pharmaceutical activity^56^.

Imran Ul-haq et al., compared PEG and LPG in in vitro and in vivo settings. They showed that LPG has an intrinsic viscosity 25 times smaller than PEG when comparing molecules of the same size. This characteristic is of high importance in formulations where higher concentrations are required. Furthermore, studies based on red blood cell (RBC) aggregation and hemolysis assays, observed that LPG did not induce any RBC aggregation even at concentrations of 10 mg/mL, while PEG induced a massive RBC aggregation at this concentration^57^. This has also been observed before for smaller molecular weight PEG and LPG^58^.

Cell-free protein synthesis was chosen to analyze fundamental characteristics of PEG- and LPG-based polymers such as impact on protein stability and integrity as well as influence on cultured human cells. The successful synthesis and modification of EPO in cell-free protein synthesis systems was shown previously^36^. In contrast to the study of 2018, we have now analyzed the effect of coupled polymers on EPOs characteristics. Interestingly, both polymers showed different behaviors after the coupling process. After conjugation of polymers, a prominent shift to higher molecular weight was visible on the SDS-PAGE. Indeed, the gel-migration looked different between both polymers even though LPG-BCN and PEG-BCN have a similar molecular weight of 10 kDa. This effect can be explained by specific interactions of the polymers within the SDS-PAGE as previously described for PEG^59^. Additionally, a comparable effect was also seen after coupling of LPG-BCN and PEG-BCN to human interleukin-4^31^. In addition to the changed gel migration, the bioconjugation of LPG and PEG showed different coupling efficiency. For PEG-BCN a coupling, efficiency of 90–95% and for LPG-BCN a coupling efficiency of 50% is estimated. These differences might occur due to specific protein-polymer interaction profiles. In the case of PEG-BCN positively charged lysines and arginines are described, whereas LPG was found in close proximity to serines and methionines^32^. The proportion of lysines and arginines is much higher in EPO in comparison to methionines and serines. Independently the coupling efficiency was sufficient for both polymers leading to a specific signal determined by autoradiography. Consequently, the effect of non-modified and modified EPO on cell culture was determined. Growth curves showed the expected results since cells only grew in presence of EPO variants. Nonetheless, differences between EPO variants were visible. Within the first three days, the growth of cells upon stimulation by non-modified and PEG- and LPG-EPO was nearly identical. After day 4, the growth of cells incubated with non-modified EPO stopped whereas cells incubated with both modified EPOs kept growing. This indicates a prolonged activity of polymer modified EPO. These findings are in agreement with described positive effects of polymer-conjugated EPO variants^7,8^.

The obtained data from the stability assay are also in agreement with previous findings. A previous study collected serum and plasma samples containing EPO and stored these samples for 14 days at different conditions. Even the sample stored at room temperature contained immunoreactive EPO after 14 days^60^.

Since cell-free protein synthesis can be performed in a µL- to liter-scale and the synthesis of pharmaceutically relevant proteins such as EPO is typically performed within a few hours the system offers a great choice for the screening of PEG alternatives coupled at pre-defined positions in human EPO.

## Supplementary Information

Supplementary Figures.

## Data Availability

All data generated or analyzed during this study are included in this published article (and its Supplementary Information files).

## Abbreviations

PEG: Poly (ethylene glycol)
LPG: Linear polyglycerol
EPO: Erythropoietin
CuAAC: Copper-catalyzed alkyne-azide cycloaddition
SPAAC: Strain-promoted azide-alkyne cycloaddition
BCN: Bicyclo[6.1.0]non-4-yne
eAzFRS: Enhanced and modified *E. coli* tyrosyl-tRNA-synthetase
AzF: P-azido-l-phenylalanine
*Sf*21: *Spodoptera frugiperda*
