# Supplementary material for: One to one comparison of cell-free synthesized erythropoietin conjugates modified with linear polyglycerol and polyethylene glycol
Source: Sci Rep. 2023 Apr 19;13:6394. doi: 10.1038/s41598-023-33463-x (PMC10115831; doi:10.1038/s41598-023-33463-x)
Supplement: Supplementary file 1 — Supplementary Figures. [file 41598_2023_33463_MOESM1_ESM.pdf]

Supplementary Material

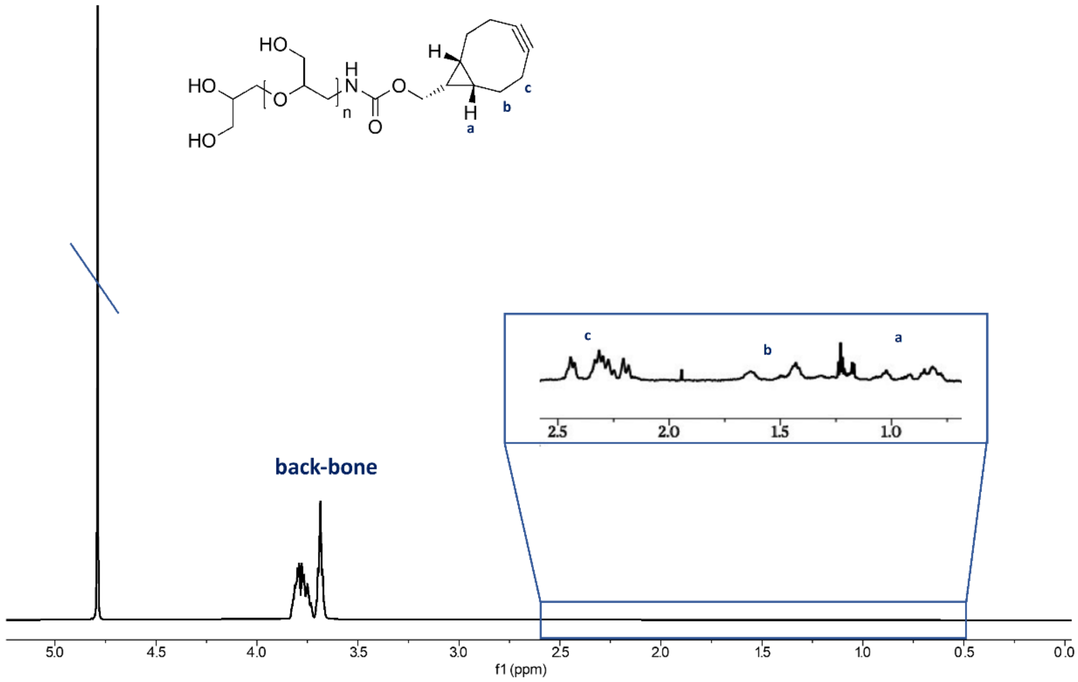

Supplementary Figure 1. 1HNMR spectrum of LPG-BCN.

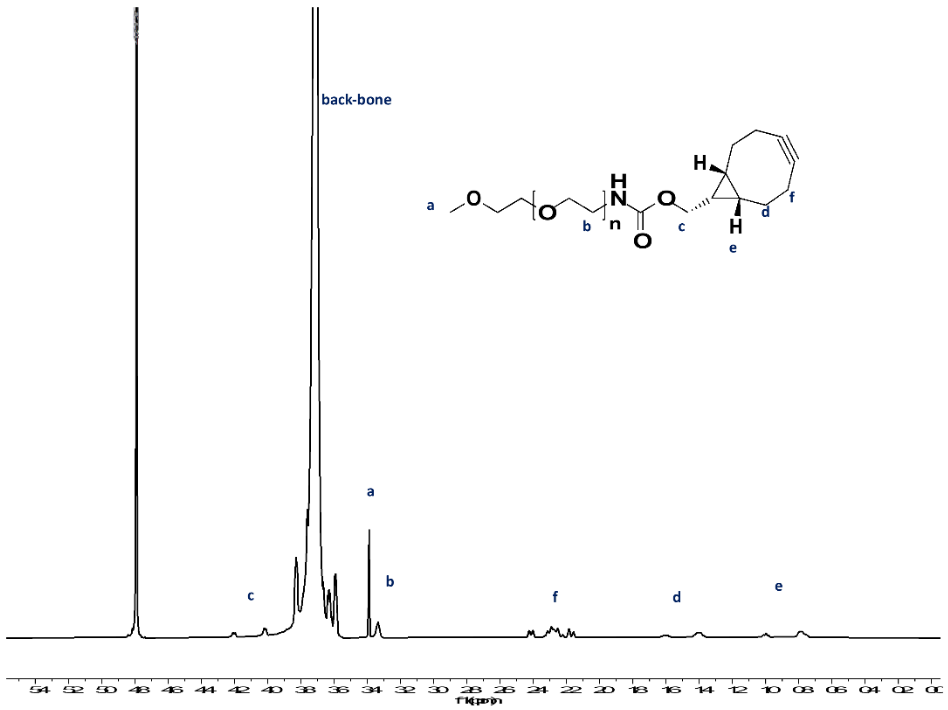

Supplementary Figure 2. ^{1}HNMR spectrum of PEG-BCN.

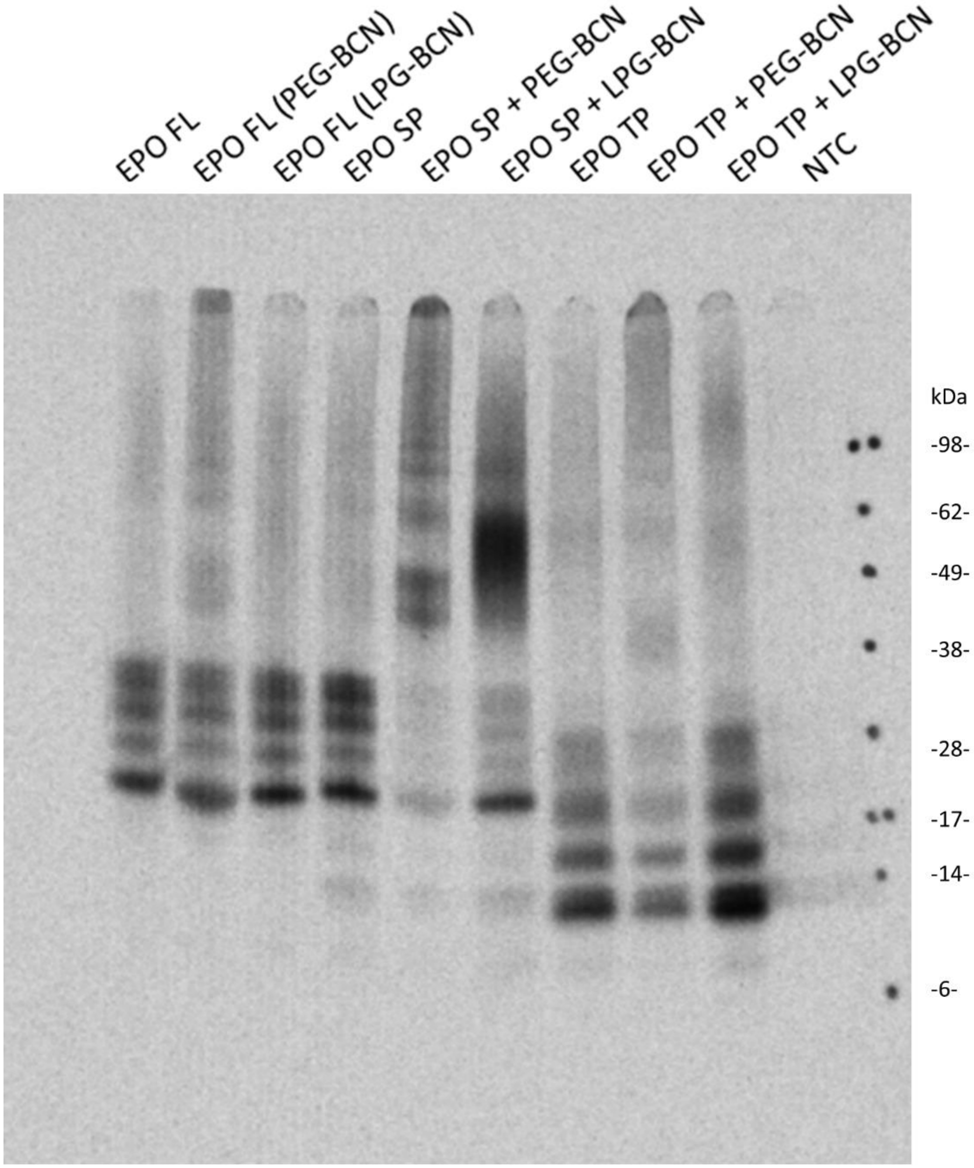

Supplementary Figure 3. Additional controls to monitor the specific coupling of polymers to Azido-EPO. Full length EPO without non-canonical amino acid (EPO FL) was incubated in absence (lane 1) and presence of PEG-BCN (lane 2) or LPG-BCN (lane 3). A slight additional band in presence of PEG-BCN was observed. Next, EPO containing the incorporated non-canonical amino acid AzF (suppression product, SP) was incubated without (Lane 4) and with PEG-BCN (lane 5) or LPG-BCN (lane 6). A clear shift of the band pattern to higher molecular weight is visible in presence of each polymer. Last, EPO was synthesized in absence of orthogonal synthetase resulting in a truncated protein at amino acid 153 (termination product, TP). There is no coupling visible in presence of PEG-BCN (lane 8) and LPG-BCN (lane 9) in comparison to the termination product without polymer (lane 7).

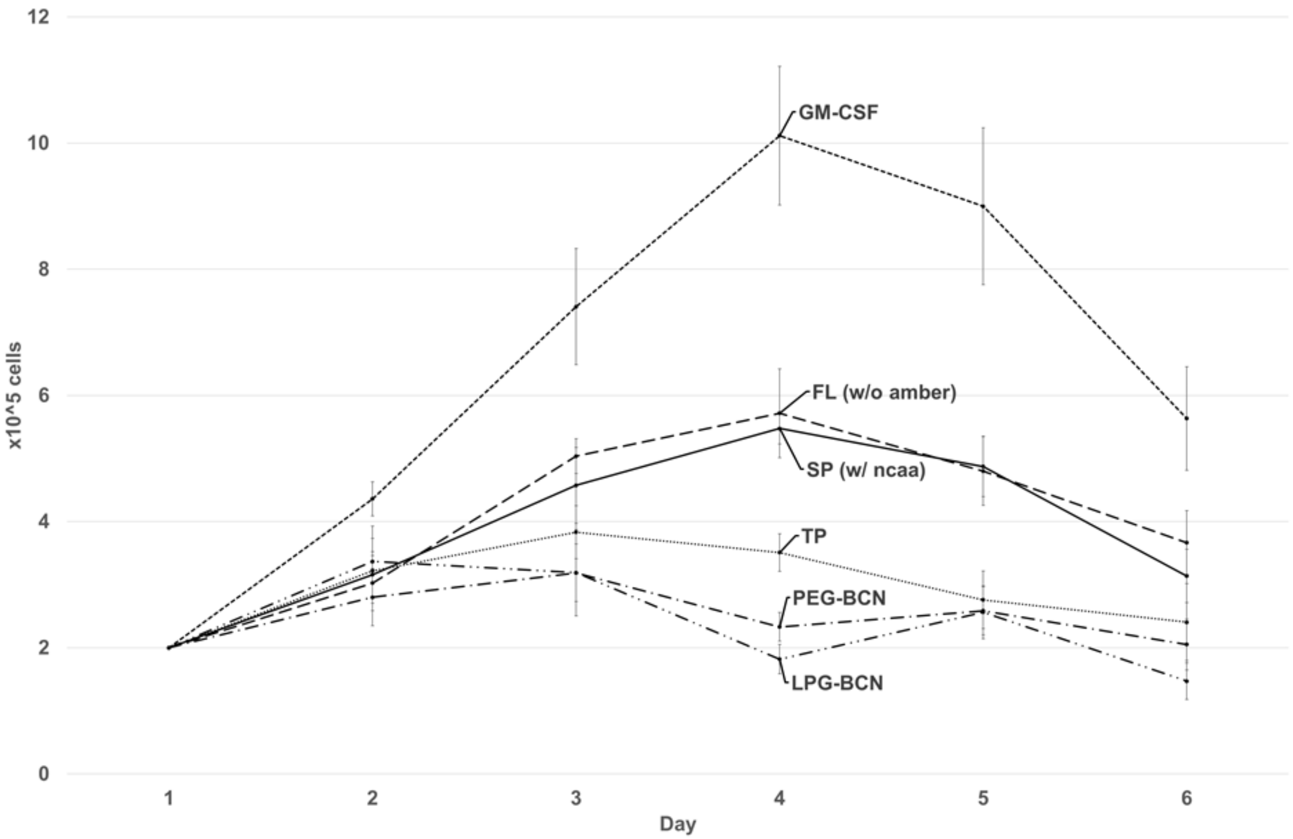

Supplementary Figure 4. Cell-based activity assay of non-modified cell-free synthesized EPO, polymer controls and GM-CSF. Growth curves of TF-1 cell-line supplemented with suppression product (continuous line), non-modified EPO (dashed line), termination product (dashed grey line), growth hormone GM-CSF (dashed line), PEG-BCN (dashed single-dot line) and LPG-BCN (dashed double-dot line). Concentrations of cell-free synthesized EPO variants were determined by TCA precipitation. 10 ng/ml of each sample was added. Cells were counted for 6 days. Data are presented as the standard deviation of three independent experiments measured in duplicates (n = 3).

Uncropped autoradiography image from main figure 2

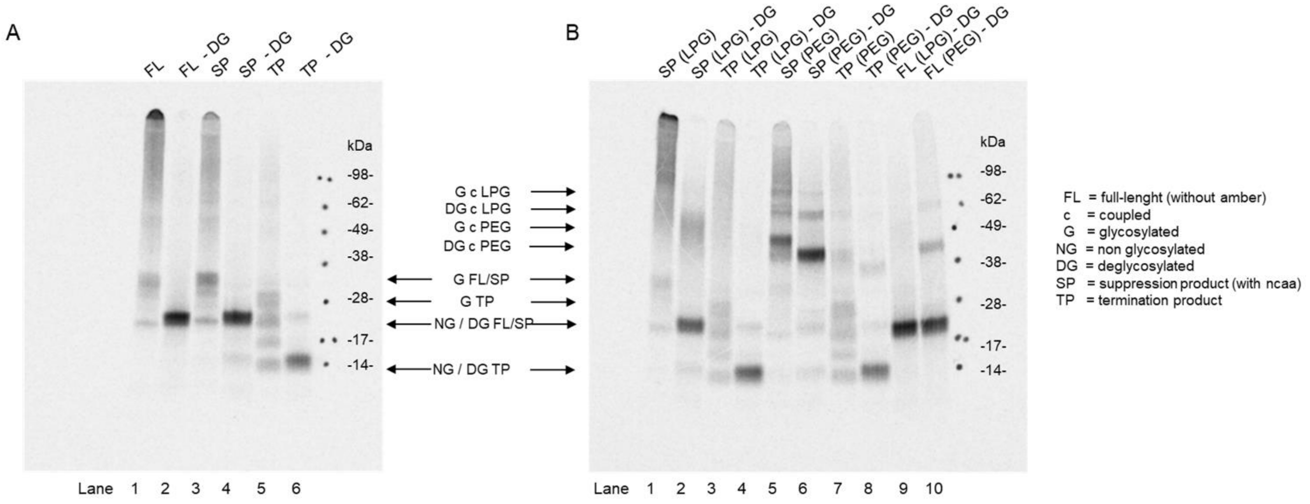

Supplementary Figure 5. Chemoselective coupling of erythropoietin with LPG-BCN and PEG-BCN. A) Autoradiography of different EPO variants: lane 1 and 2 full-length EPO without non-canonical amino acid, lane 3 and 4 suppression product with incorporated p-azido-L-phenylalanine, lane 5 and 6 termination product terminated at the amber stop codon. Each variant was analyzed in absence (lane 1, 3, 5) and presence (lane 2, 4, 6) of glycosidase PNGase F. Successful incorporation of AzF led to a comparable band pattern as seen for the full-length EPO without non-canonical amino acid. Synthesis of EPO (with amber) in absence of eAzFRS led to band pattern at a reduced molecular weight as expected for the truncated product. B) Autoradiography after coupling of LPG-BCN and PEG-BCN to EPO. Lane 1-4 and lane 9 show the coupling of LPG-BCN to AzF containing EPO (lane 1-2), terminated EPO (lane 3-4) and full-length EPO (lane 9) in absence (lane 1 and 3) and presence (lane 2, 4, 9) of PNGase F. The successful coupling of LPG-BCN to AzF containing EPO is seen by a shift of EPO corresponding bands to a higher molecular weight (lane 1 and 2) at around 50-60 kDa. Lane 5-8 and lane 10 show the coupling of PEG-BCN to AzF containing EPO (lane 5-6), terminated EPO (lane 7-8) and full-length EPO (lane 10) in absence (lane 5 and 7) and presence (lane 6, 8, 10) of PNGase F. The successful coupling of PEG-BCN to AzF containing EPO is seen by a shift of EPO corresponding bands to a higher molecular weight (lane 5 and 6) at around 40-48 kDa.

Uncropped autoradiography image from main figure 5

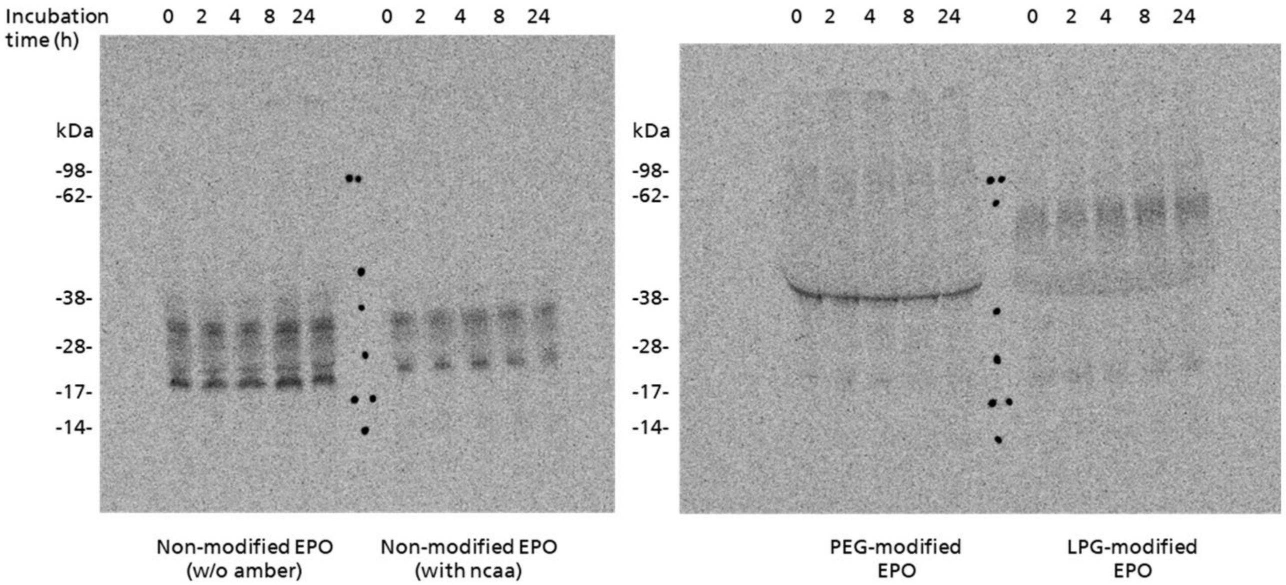

Supplementary Figure 6. Stability analysis of modified and non-modified EPO. Unmodified full-length EPO, unmodified EPO harboring a non-canonical amino acid (ncaa), PEG-modified and LPG-modified EPO were incubated up to 24 h in human serum. Afterwards samples were acetone precipitated and analyzed via SDS-PAGE with following autoradiography. No change in band pattern was seen after 24 h indicating stable EPO samples.
